# No Significant Association Between Herpes Zoster and Breast Cancer: A German Outpatient Study with over 120,000 Participants

**DOI:** 10.3390/clinpract14060218

**Published:** 2024-12-23

**Authors:** Vedanth D. Krishnan, Niklas Gremke, André Hajek, Karel Kostev, Matthias Kalder

**Affiliations:** 1 Department of Gynecology and Obstetrics, University Hospital, Philipps-University Marburg, Baldingerstraße, 35043 Marburg, Germany; 2 Department of Health Economics and Health Services Research, University Medical Center Hamburg-Eppendorf, Hamburg Center for Health Economics, 20251 Hamburg, Germany; 3 Epidemiology, IQVIA, Main Airport Center, Unterschweinstiege 2–14, 60549 Frankfurt, Germany

**Keywords:** breast cancer, herpes zoster, outpatient care research

## Abstract

Purpose: The purpose of this study was to investigate a possible association between Herpes Zoster (HZ) and the subsequent diagnosis of breast cancer (BC). Methods: Utilizing the Disease Analyzer database, anonymized medical records from German office-based practices were accessed. Longitudinal data of female patients aged 18 years and above diagnosed with HZ between 2005 and 2021 were included. Individuals without HZ diagnoses were matched to HZ patients using a nearest neighbor propensity score matching (1:1) based on age, sex, index year, average yearly consultation frequency during the follow-up, and predefined co-diagnoses. The incidence of BC in the cohort with and without HZ was evaluated using Kaplan–Meier curves and compared using the log-rank test. Finally, a univariable Cox regression analysis was conducted to assess the association between HZ and BC. Results: This study included 64,255 women with HZ and an equal number without HZ, with comparable characteristics in terms of age, visit frequency, and comorbidities. Analysis revealed no significant association between HZ and subsequent BC incidence, with similar rates observed in both HZ and non-HZ cohorts across different age groups. Conclusions: In this retrospective cohort study consisting of well-matched patients, the results indicate no significant association between an HZ infection and the development of BC over a 10-year follow-up period. This is the largest study of its kind to date.

## 1. Introduction

Herpes Zoster (HZ) results from the reactivation of the Varicella Zoster virus, typically harbored in spinal or cranial nerve ganglia [1]. The incidence of HZ infections is notably higher in individuals aged 65 and above, with rates ranging from 3.9 to 11.8 per 1000 persons per year, and is particularly prevalent among patients with a compromised immune system [2]. In 2017, a study conducted by Schroder et al. reported a nearly twofold increase in HZ incidence among immune-compromised individuals compared to those with robust immune systems. Additionally, female gender is recognized as a risk factor for HZ [3].

Breast cancer (BC) ranks as the most frequently diagnosed malignancy and the second leading cause of cancer-related mortality among women globally [4]. Its prevalence escalates with age, with 95% of new cases occurring in women aged 40 and older, typically diagnosed at an average age of 61. Given its insidious development and propensity for lymphatic dissemination, early diagnosis significantly improves survival rates. Numerous studies have highlighted a heightened incidence of HZ among cancer patients [5,6]. Conversely, studies examining the risk of first-time cancer diagnosis post-HZ yield disparate conclusions. While a Taiwanese cohort study from 2012 found no increased cancer risk associated with HZ in the general population, subsequent research, including a 2021 retrospective cohort study, noted a higher risk of hematologic cancers among HZ patients but a negative correlation with solid tumors [7,8,9]. Despite varied findings, several studies demonstrate an elevated cancer risk following HZ [10,11,12]. Finally, a retrospective study conducted by Buntix et al. in 2014 observed significant correlations between HZ and cancer risk across all women, those aged 65 and above, for specific cancer subgroups, including breast and colorectal cancer [13].

However, none of the aforementioned studies explicitly investigate the risk of breast cancer following HZ, encompassing various tumor types. Given the overlapping age of onset for BC and HZ in women, as well as the high prevalence of both HZ and BC in Germany, a specific examination of their association is warranted and highly relevant. Understanding any potential connection between HZ and BC can provide valuable epidemiological insights. This can help in identifying women who may be at an increased risk of developing breast cancer following a HZ infection, enabling targeted surveillance and early intervention. Hence, this study aims to assess the association between HZ and subsequent BC diagnosis.

## 2. Methods

### 2.1. Database

This retrospective cohort study was based on data from the Disease Analyzer (DA) database (IQVIA). This database, which has been used in several previous studies focusing on BC [14,15], contains anonymous data on diagnoses and prescriptions, as well as basic medical and demographic data from computer systems used in office-based gynecology practices [16]. The database covers approximately 3–5% of all office-based practices in Germany. The sampling method for the DA database uses statistics from the German Medical Association to determine the panel design according to the specialist group, German federal state, community size category, and age of the physician. It has previously been shown that the panel of practices included in the DA database is representative of general and specialized practices in Germany [16].

### 2.2. Study Population

**Figure 1:**
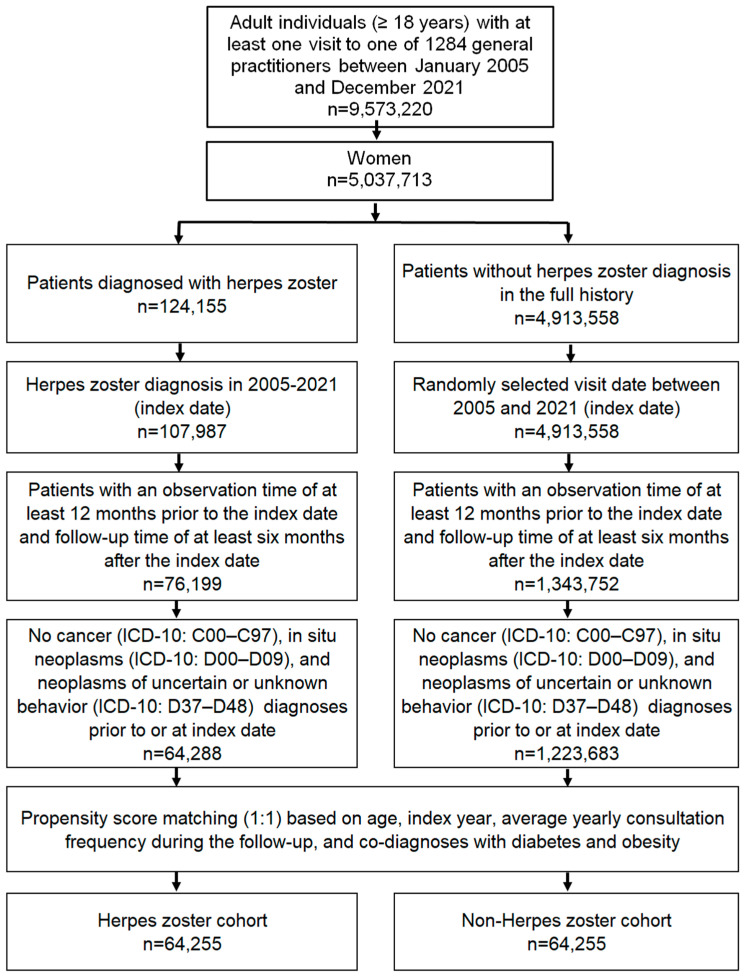
Selection of study patients.

This study included female patients aged ≥ 18 years with an initial HZ diagnosis (ICD-10: B02) in 1284 general practices in Germany between January 2005 and December 2021 (index date; Figure 1). Further inclusion criteria were an observation time of at least 12 months prior to the index date and a follow-up time of at least six months after the index date. Patients with diagnoses of cancer (ICD-10: C00–C97), in situ neoplasms (ICD-10: D00–D09), and neoplasms of uncertain or unknown behavior (ICD-10: D37–D48) prior to or at the index date were excluded.

After applying similar inclusion criteria, individuals without HZ diagnoses were matched to HZ patients using a nearest neighbor propensity score matching (1:1) based on age, sex, index year, average yearly consultation frequency during the follow-up, and co-diagnoses of diabetes (ICD-10: E10–E14) and obesity (ICD-10: E66). For the non-HZ cohort, the index date was that of a randomly selected visit between January 2005 and December 2021 (Figure 1). This study allowed a standardized mean difference in matching of less than 0.1, indicating that adequate covariate balance has been achieved.

### 2.3. Study Outcomes and Statistical Analyses

The outcomes of this study were the initial diagnoses of BC (ICD-10: C50) in up to ten years following the index date as a function of HZ. Differences in the sample characteristics between HZ and non-HZ cohorts were compared using the Wilcoxon signed-rank test for continuous variables, the McNemar test for categorical variables with two categories, and the Stuart–Maxwell test for categorical variables with more than two categories. The incidence of BC in the cohort with and without HZ was evaluated using Kaplan–Meier curves and compared using the log-rank test. Finally, a univariable Cox regression analysis was conducted to assess the association between HZ and BC. Results of the Cox regression model are displayed as hazard ratios (HRs) and 95% confidence intervals (CIs). Additionally, Cox regression analyses were conducted separately for four age groups. *p*-values of <0.05 were considered to be statistically significant. Analyses were carried out using SAS version 9.4 (SAS Institute, Cary, NC, USA).

## 3. Results

### 3.1. Basic Characteristics of the Study Sample

The present study included 64,255 women with HZ and 64,255 women without HZ. The basic characteristics of study patients are displayed in Table 1. The mean age was 59.8 years. Patients visited their general practitioners an average of 8.1 times per year during the follow-up. Due to the matched pairs design, no significant differences were observable between both cohorts in terms of age, visit frequency, and comorbidities (Table 1).

### 3.2. Association of HZ with Subsequent Breast Cancer

**Figure 2:**
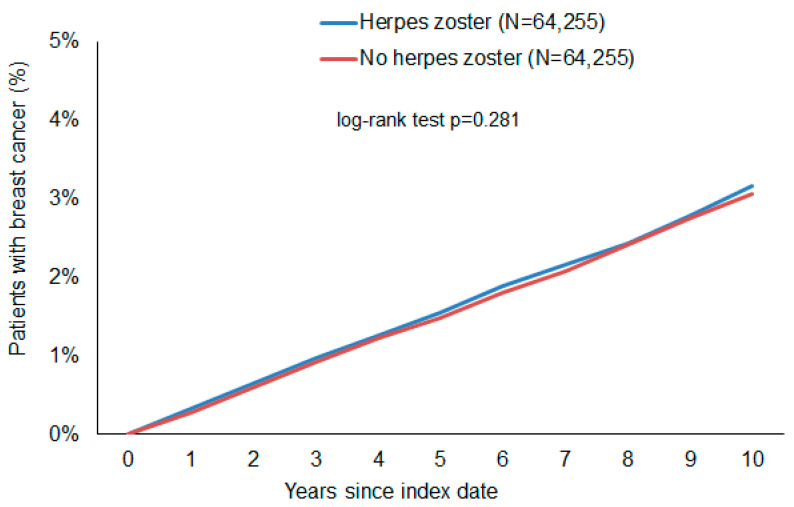
Cumulative incidence of breast cancer in female patients with and without herpes zoster.

Figure 2 shows Kaplan–Meier curves without a visible difference between the curves of HZ and non-HZ patients. The log-rank test was not significant (*p* = 0.281, chi-squared value: 1.16, degree of freedom: 1). Mean and median time to the event on the Kaplan–Meier curve was 3.9/3.1 years in the HZ cohort and 3.7/2.9 in the non-HZ cohort.

In the HZ group, this study observed a slightly increased incidence of BC (3.23 cases per 1000 patient years) as compared to the cohort without HZ (3.06 cases per 1000 patient years). However, the difference was not significant in any of the analyses that were applied. In the regression analysis, there were no significant associations between HZ and subsequent BC (HR: 1.05; 95% CI: 0.96–1.15). Age-stratified analyses also did not show a significant association (Table 2).

Kaplan–Meier curves show proportions of women with a breast cancer diagnosis in the time up to 10 years after the index date. A *p*-value of 0.281 by the long-rank test means that there is no difference between women with and without Herpes Zoster in the probability of subsequent breast cancer.

## 4. Discussion

This study revealed that there is no statistically significant association between HZ and the occurrence of BC. The number of patients (n = 128,510) included in this study makes it, to our knowledge, the largest study to date exploring the association between HZ and BC in women. Based on the results and considering absolute numbers, women over 50 years of age with a history of HZ developed cancer more often than women without HZ. Interestingly, patients below 50 years of age with HZ showed a lower incidence of BC than their control groups. This is a rare finding, as other studies have shown a higher incidence rate of BC among HZ patients compared to their control groups across all ages. These findings are based on absolute numbers but lack statistical significance.

Cotton et al. [11] conducted a prominent study on this topic in 2013, reporting a HR specifically for BC patients of 2.74. Our findings regarding the incidence rate of BC in HZ patients (HR = 1.05 among all age groups) were lower in comparison, despite our study’s much larger sample size (64,255 female patients with HZ, compared to 7803). Another study by Buntix and colleagues [13] in 2014 found a statistically significant association between HZ and BC (HR = 2.14 at 95% CI [1.11–4.12]); however, they only studied women aged > 65 years. In the same year, Chiu et al. [17] found a similar hazard ratio (HR = 1.21) to ours for female HZ patients with subsequent BC. However, they suggested the possibility of detection bias in their cohort study, as patients with HZ were more likely to be screened for subsequent malignancy.

One of the earliest findings on HZ as a risk factor for subsequent BC was published in 1955 by Wyburn-Mason [18], who noted that when HZ affected the skin of the breast, the underlying tissues were often painful and inflamed, and in some cases, galactorrhea occurred. He found that in 15 cases, after an interval ranging from three months to three and a half years, during which evidence of nervous irritation was usually present in the involved area, malignant changes developed in the breast beneath. Naturally, these findings were based on observations of a few cases instead of population cohorts and lacked screening possibilities due to the limitations of the time when the paper was written. Fortunately, the idea of HZ associated with BC became more prominent, and since then, several studies on this topic have been published. In 1982, Raggozino et al. [19] determined an increased relative risk for subsequent cancer following HZ. These findings were based on the association of HZ with immunosuppression, which increases the risk of BC. In an earlier publication this year that studied the association of mastitis and subsequent BC [20], we described how tumor microenvironments can be developed and maintained through inflammation and/or immunosuppression. Furthermore, a recent study by Tie and colleagues [21] shows that immunosuppressive cells mediate tumor progression and immune escape of cancers. In 1996, Arvin [22] concluded that the incidence of HZ increased with age or immunosuppression. Therefore, immunosuppression itself may be interpreted as the basis for the occurrence of both HZ and malignancies.

The body of published resources examining the correlation between HZ and subsequent BC is relatively sparse and limited. However, there are informative publications on HZ associated with other cancer types as well as increased cancer risk in general. Sim et al. [23] found varied incidences of specific cancers following HZ. Particularly, cancers of the lips, mouth, pharynx, and digestive or respiratory systems had a decreased incidence rate, whereas cancers of the thyroid gland, endocrine gland, and lymphoid or haematopoietic systems showed an increased incidence. The overall risk of cancer was slightly lower in the HZ group than in the non-HZ group (HR = 0.94). Similar findings were published by Liu and colleagues [24] regarding lymphoid malignancies in Taiwanese subjects. In their population-based matched-control study, the cumulative incidence of lymphoid malignancies was higher in patients with HZ (HR = 1.82). Sørensen et al. [25] examined the risk of cancer in a follow-up cohort of patients hospitalized for HZ. They found an increased frequency of several types of cancer after hospitalization for HZ, especially within the first year of follow-up. Notably, during the first year of follow-up, the relative risk (RR) was 1.3 for all malignant neoplasms and 0.8 specifically for breast cancer. In comparison, the RR beyond the first year of follow-up was 1.1 for all malignant neoplasms and 1.1 for breast cancer. Even though there was a strong association between HZ and haematological cancers, no strong association was found between HZ and BC. This data is consistent with our results, where no statistically significant association was found.

Coinciding with previous studies, this retrospective cohort study suggests that there is no benefit to performing BC screenings in patients with HZ.

## 5. Strengths and Limitations

This retrospective cohort study on the association between HZ and subsequent BC has multiple strengths. This study includes a large number of participants (n = 128,510), which enhances the statistical power and reliability of the findings. Utilizing the DA database ensures a broad and varied sample of the population since it represents general practices across all of Germany. Furthermore, the study has sufficient follow-up periods of at least six months, which allow for the assessment of long-term outcomes. This is crucial for identifying the potential delayed effects of HZ on BC incidence. The use of nearest neighbor propensity score matching to create comparable cohorts minimizes confounding variables and ensures comparability in terms of age, sex, index year, average yearly consultation frequency during the follow-up, and co-diagnoses with diabetes and obesity. Lastly, the employment of multiple statistical methods provides a thorough examination of the data and strengthens the validity of the conclusion.

However, this study is subject to certain methodological limitations. Given the nature of a retrospective cohort study, it is susceptible to biases regarding the quality of treatment or completeness of data. For example, general practitioners may have performed different treatment protocols for HZ patients and non-HZ patients, possibly resulting in performance bias. Nonetheless, since HZ does not primarily affect the breast, this is unlikely. Additionally, the database does not contain clinical data on the severity of HZ, patient histories and risk factors, or details on hospitalization and mortality. These data would enable a more detailed analysis, for instance, the ability to explore possible correlations between severity and hospitalization or whether BC is more likely to occur in such cases. These factors could provide deeper insight into the relationship between HZ and BC. Furthermore, the database used does not contain data on breast cancer staging or types. Finally, no data are available on socioeconomic status and lifestyle-related risk factors, which, especially data on smoking behavior, would be relevant in the present analysis.

## 6. Conclusions

In summary, the results indicate no significant association between HZ and subsequent BC in total cohorts as well as in different age groups. However, further research could be valuable to explore whether specific subpopulations, such as those with repeated HZ episodes or immune-compromising conditions, may experience different outcomes. Additionally, future studies may investigate the relationship between HZ and other cancer types, as any association may vary depending on the cancer in question.

## Figures and Tables

**Table 1:** Baseline characteristics of the study sample (after propensity score matching).

Variable	Proportion Among Individuals with Herpes Zoster (%) N = 64,255	Proportion Among Individuals Without Herpes Zoster (%) N = 64,255	*p*-Value
Age (Mean, SD)	59.8 (17.5)	59.8 (17.5)	0.667
Age ≤ 50	28.3	28.5	0.811
Age 51–60	20.3	20.3	
Age 61–70	20.1	19.9	
Age > 70	31.3	31.3	
Number of physician visits per year during the follow-up (Mean, SD)	8.1 (3.8)	8.1 (3.8)	1.000
Diagnoses documented within 12 months prior to or at index date			
Diabetes	16.4	16.4	1.000
Obesity	11.8	11.8	1.000

Proportions of patients in % given, unless otherwise indicated. SD: standard deviation.

**Table 2:** Association between Herpes Zoster and subsequent breast cancer in patients followed in general practices in Germany (univariable Cox regression models).

	Incidence (Cases per 1000 Patient-Years) in the Herpes Zoster Cohort	Incidence (Cases per 1000 Patient-Years) in the Non-Herpes Zoster Cohort	HR (95% CI)	*p*-Value
Total	3.23	3.06	1.05 (0.96–1.15)	0.282
Age ≤ 50	1.41	1.55	0.87 (0.69–1.11)	0.263
Age 51–60	3.10	3.00	1.03 (0.84–1.25)	0.806
Age 61–70	4.41	3.74	1.18 (0.99–1.41)	0.053
Age > 70	4.36	4.24	1.03 (0.89–1.19)	0.705

## Data Availability

Anonymized raw data are available upon reasonable request.
